# Variations in the distance between the cricoid cartilage and targets of stellate ganglion block in neutral and extended supine positions: an ultrasonographic evaluation

**DOI:** 10.1007/s00540-016-2236-8

**Published:** 2016-08-30

**Authors:** Jiwon An, Youn-Woo Lee, Woo Young Park, Sungchul Park, Hyungbae Park, Ji Won Yoo, Jong Bum Choi

**Affiliations:** 1 Department of Anesthesiology and Pain Medicine, Anesthesia and Pain Research Institute, Gangnam Severance Hospital, College of Medicine, Yonsei University, Seoul, Korea; 2 Department of Anesthesia, Sheikh Khalifa Specialty Hospital, Ras Al Khaimah, United Arab Emirates; 3 Department of Anesthesiology and Pain Medicine, College of Medicine, Seoul National University, Seoul, Korea; 4 Department of Anesthesiology and Pain Medicine, College of Medicine, Ajou University, San 5, Wonchon-dong, Yeongtong-gu, Suwon, 443-721 Korea; 5 Department of Internal Medicine, School of Medicine, University of Nevada, Las Vegas, NV USA

**Keywords:** Cricoid cartilage, Transverse process, Stellate ganglion block

## Abstract

**Purpose:**

Anatomic variations complicate surface landmark-guided needle placement, thereby increasing nerve blockade failure rate. However, little is understood about how anatomic distances change under different clinical conditions. As the cricoid cartilage is an easy and accurate landmark, we investigated changes in distance between the sixth or seventh cervical transverse processes (C6TP or C7TP) and the cricoid cartilage in neutral and extended supine positions.

**Methods:**

Forty-two patients (16 men, 26 women) were included in this study. Distances between the cricoid cartilage and C6TP/C7TP were measured using ultrasonography with the patient in neutral and extended supine positions.

**Results:**

C6TP and C7TP were caudally located at 6.0 ± 8.1 and 15.1 ± 7.2 mm, respectively, from the cricoid cartilage in the neutral supine position, and at 15.2 ± 8.0 and 25.3 ± 8.0 mm, respectively, in the extended supine position. In the extended supine position, the cricoid cartilage was more cephalad than C6TP and C7TP in all patients. The distance from the cricoid cartilage to C6TP was 12.1 ± 7.6 mm in men and 17.2 ± 7.7 mm in women.

**Conclusion:**

C6TP and C7TP are located approximately 15 and 25 mm, respectively, caudal to the cricoid cartilage in the extended supine position. Our results highlight the fact that there can be significant anatomic variation between the extended and neutral supine positions used in stellate ganglion block, which should be kept in mind when devising easily identifiable and palpable surface landmarks.

## Introduction

Stellate ganglion block (SGB) is a sympathetic nerve block used in the treatment of head and neck pain, pain of the upper extremities, vascular disease, or sensorineural hearing loss [1]. SGB is generally performed via the anterior paratracheal approach at the level of the C6 transverse process (C6TP) and C7TP. Although ultrasound or fluoroscopy-guided approaches are available, the classical blind technique is mostly used in primary pain clinics, because ultrasound or fluoroscopy-guided techniques require training and are expensive. Moreover, the fluoroscopy-guided technique is injurious to health because of exposure to radiation. However, the classical blind technique is associated with risks due to inappropriate needle placement, such as carotid trauma, neural injury, and vascular trauma. The most common classical blind technique used for SGB is the anterior paratracheal approach at the level of the C6TP [2]. In this approach, the patient is placed in the supine position with a small pillow between the shoulders. After skin draping, the index and middle fingers are placed between the medial head of the sternocleidomastoid muscle and the trachea. A 22-gauge needle is directed through the skin in a perpendicular direction until bone is encountered at the level of the sixth or seventh cervical vertebrae [3]. In this technique, the most important landmark is the C6TP, also known as Chassaignac’s tubercle. It is widely known that, in most patients, the C6TP is located lateral to the cricoid cartilage and is easily palpated [2]. Hence, some clinicians use the cricoid cartilage as the landmark for finding the C6TP. However, in most patients, the C6TP is not located lateral to the cricoid cartilage while performing SGB, because SGB is performed in the extended supine position with a pillow under the patient’s neck, and the cricoid cartilage is expected to be displaced cephalad [4]. Thus, we assume that the cricoid cartilage could be displaced, but it is not clear from textbooks or any research that the cricoid cartilage will displace when the neck extends. Furthermore, it is not known what distances the cricoid cartilage will displace when the neck extends. In this study, we measured the distance from the C6TP and C7TP to the cricoid cartilage in the neutral supine position and the extended supine position using ultrasonography.

## Materials and methods

**Fig. 1:**
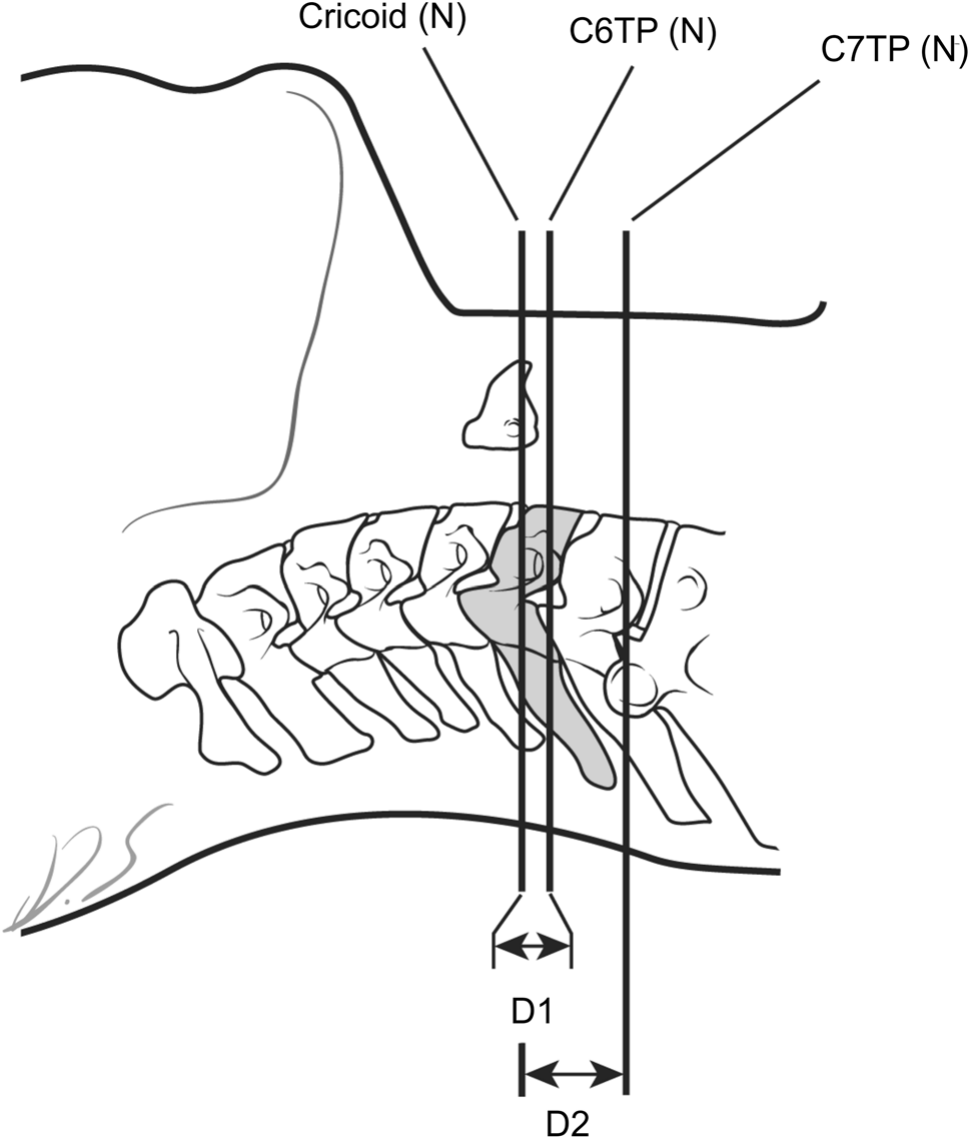
Schematic of the neutral supine position of the neck. Cricoid(N) is the line perpendicular to the ground at the level of the middle point of the cricoid cartilage on the anterior neck surface in the neutral supine position. C6TP(N) is the line perpendicular to the neck and parallel to Cricoid(N), passing through the midpoint of the C6 transverse process in the anterolateral ultrasonographic view in the neutral supine position. C7TP(N) is the line perpendicular to the neck and parallel to the line Cricoid(N), passing through the midpoint of the C7 transverse process in the anterolateral ultrasonographic view in the neutral supine position. *D1* is the distance between Cricoid(N) and C6TP(N). *D2* is the distance between Cricoid(N) and C7TP(N)

**Fig. 2:**
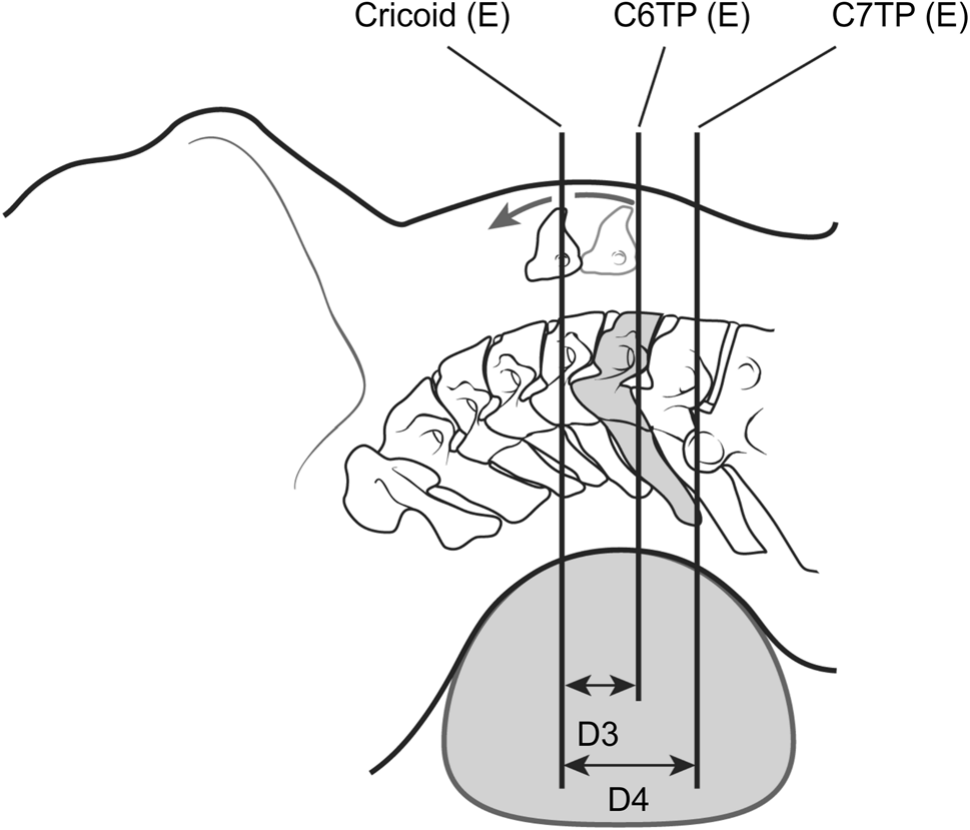
Schematic of the extended supine position of the neck. Cricoid(E) is the line perpendicular to the ground at the level of the middle point of the cricoid cartilage on the anterior neck surface in the extended supine position. C6TP(E) is the line perpendicular to the neck and parallel to the line Cricoid(E), passing through the midpoint of the C6 transverse process in the anterolateral ultrasonographic view in the extended supine position. C7TP(E) is the line perpendicular to the neck and parallel to the line Cricoid(E), passing through the midpoint of the C7 transverse process in the anterolateral ultrasonographic view in the extended supine position. *D3* is the distance between Cricoid(E) and C6TP(E). *D4* is the distance between Cricoid(E) and C7TP(E)

This study was approved by the institutional review board at Yonsei University, Gangnam Severance Hospital. Forty-two patients were enrolled from November 2011 to October 2012 at the pain clinic in our hospital. Written informed consent was obtained from all patients for inclusion in this study. Patients aged >20 years and undergoing musculoskeletal ultrasonography were included. Exclusion criteria were previous neck or cervical spine surgery; a history of rheumatoid arthritis, cerebral palsy or tumor; severe pain at the time of evaluation; and illiteracy. After giving informed consent, patients were placed in the supine position with the neck in the neutral position. The anterior portion of the neck was scanned using ultrasonography (S-nerve; SonoSite Inc., Bothell, WA, USA) with a linear probe drawn longitudinally along the axis of the cervical spine until the cricoid cartilage was located downward from the thyroid cartilage. In this view, we marked the center of the cross-section of the cricoid cartilage and drew a line perpendicular to the neck; this was labeled ‘Cricoid(N)’. In the transverse view of the neck, the neck was scanned anterolaterally with the linear probe along the axis of the neck. After locating the C6TP and C7TP, we marked the two midpoints of the transverse processes on the skin and drew lines perpendicular to the neck and parallel to the line Cricoid(N); these were labeled ‘C6TP(N)’ and ‘C7TP(N)’. The distance between Cricoid(N) and C6TP(N) and that between Cricoid(N) and C7TP(N) was then measured (Fig. 1). To establish the extended supine position, we placed a 10-cm diameter cylindrical pillar under the neck, extending it. A pain clinician with 5 years of experience adjusted the neck until the standard SGB position was reached. Repeating the procedure for the neutral supine position, we drew parallel lines passing through the center of the cross-sections of the cricoid cartilage, C6TP, and C7TP and perpendicular to the neck, and labeled these lines as Cricoid(E), C6TP(E), and C7TP(E), respectively (Fig. 2). We measured the distance between Cricoid(E) and C6TP(E) and that between Cricoid(E) and C7TP(E). If the cricoid cartilage was positioned more cephalad than C6TP or C7TP, we assigned a positive value to the distance; if it was more caudal than C6TP or C7TP, we gave a negative value to the distance.

The requisite patient sample size was determined from the raw data of a previous study by power analysis, with an *α*-error of 0.05 and a *β*-error of 0.2. The mean sample size (standard deviation) was −23.3 (9.6), −5.0 (12.5), −5.2 (10.4), and 14.4 (11.8) in the neutral position in men, extended position in men, neutral position in women, and extended position in women, respectively [4]. The final sample size was determined to be 42 (16 men, 26 women), allowing for a dropout rate of 10 %.

Patient characteristics are reported using descriptive statistics. We calculated means and standard deviations for distances from the cricoid cartilage to the C6TP and C7TP in the neutral and extended positions. The paired *t* test was used to compare differences between the extended and neutral positions. The two-sample *t* test was used to compare differences by sex. A *p* value <0.05 was considered to be statistically significant. Statistical Package for the Social Sciences version 20.0 software (IBM Corp., Armonk, NY, USA) was used for all statistical analyses.

## Results

Forty-two patients (16 men, 26 women; mean age 54.5 years; range 27–91 years) were included in the study. Demographic data for the participants are shown in Table 1.

**Table 1:** Demographic data of the patients

	Male	Female	Total
No.	16	26	42
Age (years)	46.1 (14.7)	59.7 (13.8)	54.5 (15.5)
Height (cm)	172.5 (9.1)	156.3 (6.0)	162.5 (10.7)
Weight (kg)	74 (10.5)	58.9 (10.1)	64.7 (12.6)

Values are mean (SD)

In the neutral supine position, the mean distances from the cricoid cartilage to the C6TP and C7TP were 6.0 and 15.1 mm, respectively. In the extended supine position, these distances were 15.2 and 25.3 mm, respectively. The difference between the corresponding distances in the neutral and extended supine positions was statistically significant (*p* < 0.001 for both; Table 2).

**Table 2:** Distances from the cricoid cartilage to the C6 transverse process or the C7 transverse process in the neutral and extended supine positions of the neck (mm)

	Neutral supine position	Extended supine position	*p* value
Cricoid-C6TP	6.0 (8.1)	15.2 (8.0)	<0.001*
Cricoid-C7TP	15.1 (7.2)	25.3 (8.0)	<0.001*

Values are mean (SD)

*Cricoid-C6TP* the distance from the cricoid cartilage to the C6 transverse process, *Cricoid-C7TP* the distance from the cricoid cartilage to the C7 transverse process

*p < 0.05 between neutral and extended position

The distance from the cricoid cartilage to the C6TP was significantly different between men and women in both the neutral (*p* = 0.028) and extended (*p* = 0.041) supine positions. The distance from the cricoid cartilage to the C6TP was significantly different between the neutral and extended supine positions in both sexes (*p* < 0.001 for men and women; Table 3).

**Table 3:** Distances from the cricoid cartilage to the C6 transverse process based on sex in the neutral and extended supine positions of the neck (mm)

	Neutral supine position	Extended supine position	*p* value
Male	2.6 (9.2)	12.1 (7.6)	<0.001*
Female	8.2 (6.7)	17.2 (7.7)	<0.001*
*p* value	0.028^†^	0.041^†^	

Values are mean (SD)

*p < 0.05 between neutral and extended position

^†^ *p* < 0.05 between men and women

The distance from the cricoid cartilage to the C7TP was not significantly different between men and women in the neutral supine position (*p* = 0.148) or in the extended supine position (*p* = 0.132); however, it was different between the two positions in both men (*p* < 0.001) and women (*p* < 0.001; Table 4).

**Table 4:** Distances from the cricoid cartilage to the C7 transverse process based on sex in the neutral and extended supine positions of the neck (mm)

	Neutral supine position	Extended supine position	*p* value
Male	13.0 (7.1)	22.9 (8.2)	<0.001*
Female	16.4 (7.2)	26.8 (7.6)	<0.001*
*p* value	0.148	0.132	

Values are mean (SD)

*p < 0.05 between neutral and extended position

## Discussion

In this study, we evaluated the distance between the cricoid cartilage and C6TP and C7TP when patients are in the neutral supine position and the extended supine position. The cricoid cartilage was positioned, on average, more cephalad than the C6TP and C7TP in the supine position in all except seven patients (16.7 %) in the neutral supine position [six of these seven patients (94.2 %) were men], and in all patients (100 %) in the extended supine position. This means that the targets of SGB, i.e., C6TP and C7TP, should be located caudal to the cricoid cartilage in the extended position.

Further research on SGB target points was performed using radiography [4]. This study analyzed the cervical vertebrae and explored the relationship between the cricoid cartilage and the C6TP. However, the X-ray of the cervical vertebrae was not obtained in the supine position, so the position was different from that used in SGB.

When the patient is placed in an extended supine position for SGB, the cricoid cartilage moves cephalad. We cannot conclude that the tubercle that moves laterally from the cricoid cartilage is the C6TP. However, we can conclude that when performing SGB with the patient in an extended supine position, C6TP and C7TP are found more caudally than the cricoid cartilage. In the neutral and extended supine positions, the distance from the cricoid cartilage to the C6TP in men was significantly different from that in women, whereas no such difference was found for the distance between the cricoid cartilage and the C7TP. Thus, the SGB injection point will be different between men and women for C6TP, but will not be different for C7TP. The reason for these results may be the anatomic difference between the sexes in the thyroid cartilage or muscular connections between the thyroid cartilage and cricoid cartilage.

There are a few limitations to this study. First, we did not explore any racial differences or effects of aging, body mass index, or other factors that could affect anatomy and neck movement. Second, we did not perform SGB to confirm its success rate using this technique.

To summarize, in this study, we evaluated the distance between the cricoid cartilage and C6TP and C7TP when patients are in the neutral supine and extended supine positions. Establishing these distances will help clinicians find the C6TP and C7TP, targets of SGB, easily and accurately. The currently used classical blind technique is easily performed by clinicians but is inconvenient for patients, especially those who are muscular or have a short neck. The method of finding the C6TP or C7TP by locating the cricoid cartilage is inaccurate at present because the cricoid cartilage moves upon neck extension. Real-time scanning by ultrasonography or fluoroscopy is expensive, time-consuming (requires training), and hazardous because of radiation exposure. Therefore, our method may provide the estimated location of C6TP or C7TP to primary pain clinicians performing SGB procedures. Further studies are required to establish the practicality and accuracy of this method by examining the outcome of SGB when the C6TP and C7TP are located using the cricoid cartilage as a landmark.
